# Supplementary material for: Serum Dickkopf-1 in Combined with CA 19-9 as a Biomarker of Intrahepatic Cholangiocarcinoma
Source: Cancers (Basel). 2021 Apr 12;13(8):1828. doi: 10.3390/cancers13081828 (PMC8069292; doi:10.3390/cancers13081828)
Supplement: Supplementary file 1 [file cancers-13-01828-s001.pdf]

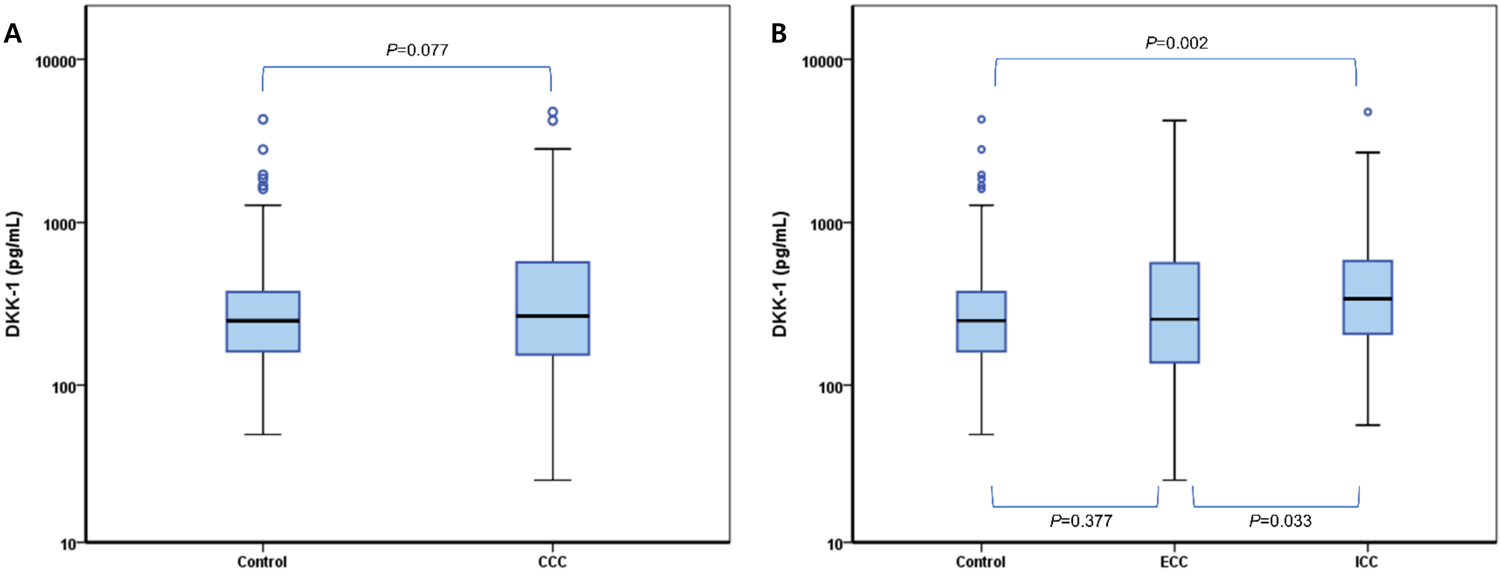

Figure S1. Serum DKK-1 level. (A) The median level of DKK-1 in the control group (n=200) and in patients with CCC (n=356) were 249.8 pg/mL and 267.2 pg/mL, respectively. (*P*=0.077) (B) When patients with CCC were divided into the ECC group (n=277) and ICC group (n=79), the median levels of DKK-1 were 255.0 pg/mL and 340.5 pg/mL, respectively, and there was a significant difference between the control group and the ICC group. (*P*=0.002) DKK-1, Dickkopf-related protein 1; CCC, cholangiocarcinoma; ECC, extrahepatic cholangiocarcinoma; ICC, intrahepatic cholangiocarcinoma.

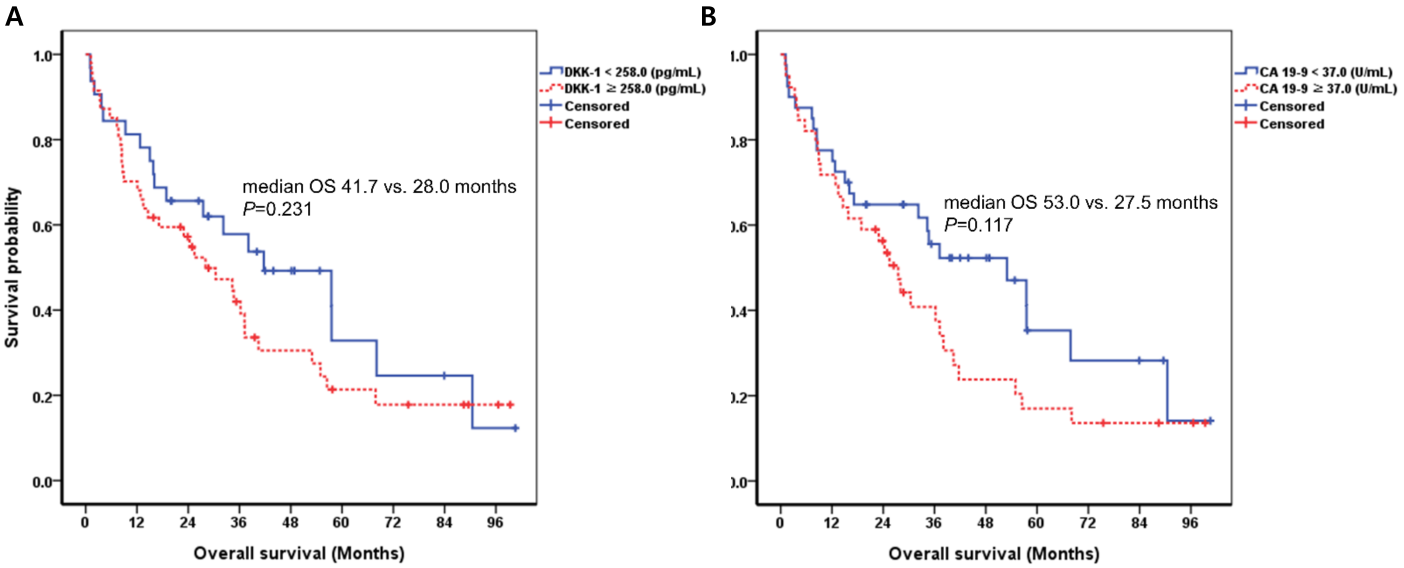

Figure S2. Correlation between DKK-1 and CA 19-9 level and the survival of patients with ICC using the Kaplan-Meier curve. The cutoff value of DKK-1 and CA 19-9 were 258.0 pg/mL and 37.0 U/mL, respectively. (A) The median survival of the low and high DKK-1 expression groups were 41.7 and 28.0 months, respectively, and there was no significant difference between the groups (*P*=0.231 by log-rank test). (B) The median survival of the low and high CA 19-9 expression groups were 53.0 and 27.5 months, respectively, and there was no significant difference between the groups (*P*=0.117 by log-rank test). DKK-1, Dickkopf-related protein 1; CA 19-9, carbohydrate antige 19-9; ICC, intrahepatic cholangiocarcinoma; OS, overall survival.

Table S1. Etiologies of cholangiocarcinoma and serum levels of biomarkers.

	Sporadic	HBV	HCV	Liver fluke	Liver cirrhosis	PSC	Bile duct stone
DKK-1 (pg/mL)	218.9	219.0	70.7	318.4	256.5	-	525.6
CA 19-9 (U/mL)	51.3	171.3	2746.0	95.4	203.7	-	89.4
OS (months)	43.4	51.9	18.9	24.3	35.2	-	36.5
n (%)	75 (78.9%)	5 (5.3%)	1 (1.1%)	1 (1.1%)	6 (6.3%)	0 (0%)	7 (7.4%)

DKK-1, Dickkopf-related protein 1; CA 19-9, carbohydrate antigen 19-9; HBV, Hepatitis B virus; HCV, Hepatitis C virus; PSC, Primary sclerosing cholangitis.

Table S2. Performance characteristics of DKK-1 and CA 19-9

	Sensitivity (%)	Specificity (%)	PPV (%)	NPV (%)	Positive LR	Negative LR
DKK-1	59.5	56.9	40.5	74.0	1.38	0.71
CA 19-9	49.4	98.8	95.1	79.8	41.17	0.51
DKK-1 plus CA 19-9	74.7	56.3	45.7	81.8	1.71	0.45

DKK-1, Dickkopf-related protein 1; CA 19-9, carbohydrate antigen 19-9; PPV, positive predictive value; NPV, negative predictive value; LR, likelihood ratio.
